# Effects of bushen huoxue method for knee osteoarthritis: A protocol for systematic review and meta-analysis

**DOI:** 10.1097/MD.0000000000020659

**Published:** 2020-06-12

**Authors:** Guocai Chen, Xiangling Ye, Yingxin Guan, Wengang Liu, Jianping Du, Nan Yao, Xuemeng Xu

**Affiliations:** a The Fifth Clinical Medical School, Guangzhou University of Chinese Medicine; b Guangdong Second Traditional Chinese Medicine Hospital; c Guangdong Provincial Key Laboratory of Research and Development in Traditional Chinese Medicine, Guangdong Province Engineering Technology Research Institute of Traditional Chinese Medicine, Guangzhou, Guangdong, P.R. China.

**Keywords:** bushen huoxue, knee osteoarthritis, meta-analysis, protocol, systematic review

## Abstract

**Background:**

Knee osteoarthritis (KOA) is a common progressive joint disorder in old people. Bushen huoxue (BSHX) is a classical method of TCM in treating KOA. However, there is no systematic review related to BSHX for KOA. The purpose of this study is to provide a comprehensive and reliable evaluation of the clinical evidence of BSHX in the treatment of KOA.

**Methods:**

We searched relevant studies on BSHX for KOA from the databases of PubMed, Embase, MEDLINE, Cochrane Library Central Register of Controlled Trials, China national knowledge infrastructure database (CNKI), Wan fang database, Chongqing VIP information, and SinoMed from their inception to May 2020. Two researchers will select and evaluate qualified studies independently. The primary outcomes of this review will focus on pain intensity. The meta-analyses will be performed by using the RevMan 5.3.

**Results:**

The study will provide a comprehensive evaluation of the efficacy and safety of the BSHX method for patients with KOA.

**Conclusion:**

The results of this systematic review will provide evidence to judge whether BSHX is an effective intervention for patients with KOA.

## 1 Introduction

Knee osteoarthritis (KOA) is one of the most common form of arthritis, affecting millions of people worldwide. It is a degenerative type of arthritis that occurs most often in old people.^[1]^ The main symptoms of KOA are knee pain and dysfunction, which affect the quality of life and impose a heavy financial burden on families and society.^[2,3]^ Moreover, KOA is considered to be the most common cause of disability among the elderly.^[4]^ According to an epidemiological survey in China, the prevalence of symptomatic KOA among the residents aged 45 years and older was 8.1%.^[5]^ As China's elderly population continues to rise, this disease becomes more and more prominent. Therefore, effective treatment of KOA is of great significance to reduce the rate of disability and improve the quality of life.

Traditional Chinese medicine (TCM), with a history of thousands of years of clinical practice, has been used to treat KOA for a long time. It is gaining more and more attention owing to the characteristics of multi-target effects and fewer side effects.^[6]^ According to TCM syndromes, shen deficiency and blood stasis are regarded as the main pathogenesis of KOA.^[7,8]^ Thus, the bushen huoxue (BSHX) method should be the principal method for the treatment of KOA. Clinical study has demonstrated that BSHX has an effect in relieving clinical symptoms and improving rheological properties, and has anti-inflammatory and anti-oxidation functions.^[9]^ Several experiments have shown that BSHX has a good effect on the therapy of KOA, and its mechanism may be related to the decreasing of nitric oxide (NO) in the serum, synovium, and joint cartilage.^[10,11]^ With the publication of several trials on BSHX for KOA, evidences have certificated that BSHX has a good clinical effect. Although BSHX is a common method in managing KOA, there is still a lack of systematic review to summarize the efficiency of BSHX in treating KOA. Hereby, the purpose of the study is to systematically review current available randomized controlled trials (RCTs) to assess the efficacy and safety of the BSHX in the management of KOA.

## 2 Material and methods

The protocol of this review has been registered with the Open Science Framework (OSF, https://osf.io/). The registration DOI of this study is 10.17605/OSF.IO/D3U6Z. We will refer to the preferred reporting items for the systematic review and meta-analysis (PRISMA) to perform this work.^[12]^

### 2.1 Inclusion criteria

#### 2.1.1 Type of studies

In this work, RCTs that explore the specific efficacy and safety of the BSHX in the treatment of KOA will be included. Non-RCTs and observational studies will not be considered.

#### 2.1.2 Types of patients

All patients with a confirmed diagnosis with KOA will be included. There will be no limitation about age, sex, region, grades, and other factors.

#### 2.1.3 Types of interventions and comparisons

Patients in the treatment group were treated with BSHX alone or in combination with conventional pharmacotherapies. Control interventions will include no treatment, placebo control, and conventional pharmacotherapies. Pharmacotherapies include drugs recommended by the international or domestic authorized clinical guidelines.

#### 2.1.4 Types of outcomes

The primary outcomes of this review will focus on pain intensity. The secondary outcomes included the self-reported function, objective performance, quality of life, and adverse events in the treatment. If >1 scale was reported for an outcome, we selected the scale that was more comprehensively reported and highest in the ranking order proposed.^[13]^

### 2.2 Search strategy

To identify all relevant studies, we will search the following databases from inception to May 2020: PubMed, Embase, MEDLINE, Cochrane Library Central Register of Controlled Trials, China national knowledge infrastructure database (CNKI), Wan fang database, Chongqing VIP information, and SinoMed. Two authors (GC and XY) will search and screen all the relevant studies independently. A search strategy that combines MeSH terms and free words will be adopted. The search strategy was as follows:

1#: Search (((((bushen huoxue[MeSH Terms]) AND bushen[Title/Abstract]) AND huoxue[Title/Abstract]) AND bushen formula[MeSH Terms]) AND tonifying kidney[Title/Abstract]) AND activating blood[MeSH Terms].
2#: Search (((Osteoarthritis, Knee[MeSH Terms]) AND Osteoarthritis[MeSH Terms]) AND knee osteoarthritis[Title/Abstract]) AND osteoarthritis of the knee [Title/Abstract]
3#: Search ((((((((randomized controlled trial[Title/Abstract]) AND RCT[Title/Abstract]) AND controlled clinical trial[Title/Abstract]) AND randomized[Title/Abstract]) AND randomly[Title/Abstract]) AND random[Title/Abstract]) AND controlled[Title/Abstract]) AND control[Title/Abstract]) AND trial[Title/Abstract].
#1 and #2 and #3

### 2.3 Study selection and data extraction

#### 2.3.1 Selection of studies

**Figure 1:**
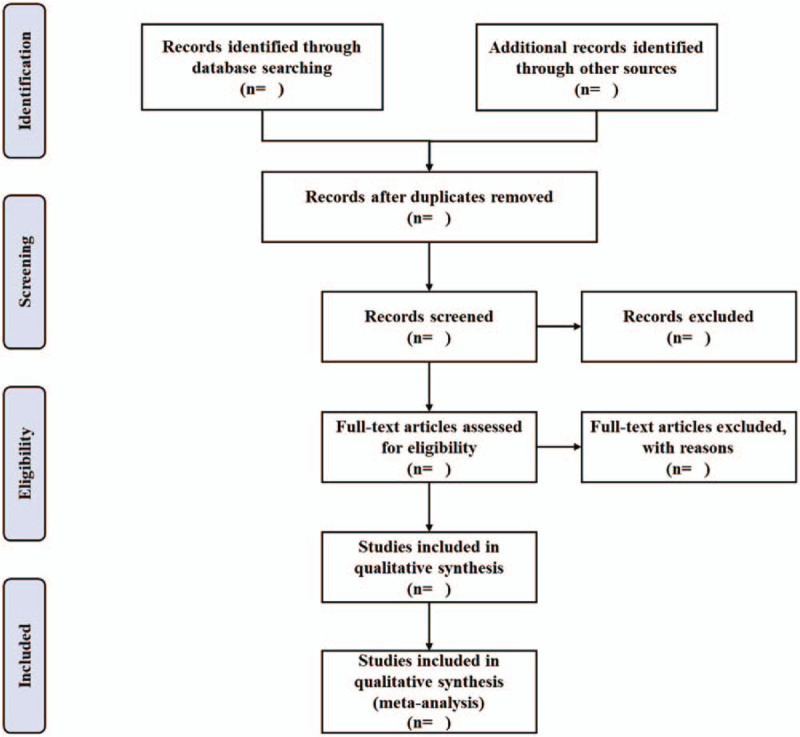
Flow chart of study selection.

Two reviewers (GC and XY) will extract the citations from the above databases by EndNote X9.0 (Stanford, Connecticut, https://endnote.com) independently. Based on the research criteria and search strategies, we will review the topics and abstracts. The eligible articles will be further determined for inclusion by reading the full text. Any different opinions generated between the 2 reviewers will be resolved through consulting another researcher. A PRISMA flow chart will be drawn to illustrate the study selection procedure (Fig. 1).

#### 2.3.2 Data extraction and management

Two researchers will extract information from the documents that met the inclusion criteria, including the first author's name, year of publication, study design, intervention, sample size, duration of intervention, and outcomes. We will contact the corresponding authors for more information if data are missing or unclear.

#### 2.3.3 Assessment of risk of bias

The Cochrane Handbook for systematic reviews of interventions (V.5.1) will be used to assess a broad category of biases. Two reviewers (GC and XY) will evaluate the methodological quality of the researches independently. The risk of bias of a trial will be evaluated through 7 items: random sequence generation, allocation concealment, blinding of participants and personnel, blinding of outcome assessment, incomplete outcome data, selective reporting, and other sources of bias. The studies will be evaluated as “Low risk,” “High risk,” or “Unclear risk.”

#### 2.3.4 Measures of treatment effect

For dichotomous data, the risk ratio (RR) with 95% CIs will be used. For continuous outcomes, the mean difference (MD) or standard mean difference (SMD) with 95% CIs will be utilized for evaluating the treatment effect.

#### 2.3.5 Assessment of heterogeneity

Before conducting a meta-analysis, we will assess study heterogeneity using the Cochrane *X*^2^ and *I*^2^ tests.^[14]^ When *P* ≥ .05 and *I*^2^ ≤ 50%, it indicates that there is no statistical heterogeneity or the heterogeneity is small. When *P* < .05 and *I*^*2*^ > 50%, it suggests that the study has significant statistical heterogeneity.

#### 2.3.6 Assessment of reporting bias

When there are >10 trials included in this review, a funnel plot and statistic test will be developed to evaluate reporting bias of the included studies. The reporting bias will be statistical appraised based on the Egger test.^[15]^

#### 2.3.7 Data synthesis

We will synthesize and analyze the data using the RevMan 5.3 software (Version 5.3, Copenhagen: The Nordic Cochrane Center, The Cochrane Collaboration, 2014) provided by Cochrane Collaboration. Heterogeneity between these studies will be tested by the *I*^2^ statistic. If *I*^2^ ≤ 50%, a fixed effect model will be used to calculate the RR and MD. If *I*^2^ > 50%, a random effect model will be applied to synthesize the data. Subgroup analysis or sensitivity analysis will be performed to explore the causes of heterogeneity. If there are >10 studies are included in the meta-analysis, a meta-regression will be conducted to explore the sources of heterogeneity.

#### 2.3.8 Subgroup analysis

If necessary, a subgroup analysis will be conducted to explain the possible causes of heterogeneity in the previous analysis. It will be conducted according to variations in the characteristics of the participants, BSHX treatments, durations, frequencies, the grade of KOA, age, sex, and controls.

#### 2.3.9 Sensitivity analysis

A sensitivity analysis will be conducted to monitor the robustness of the results. By excluding studies with small sample sizes, weak methodologies, and data loss, the meta-analysis will be repeated. In this way, we will be able to assess the impact of these studies on the overall results and whether the results are reliable.

#### 2.3.10 Grading the quality of evidence

The quality of evidence for all outcomes will be evaluated using the Grading of Recommendations Assessment, Development, and Evaluation (GRADE).^[16]^ In the GRADE system, the quality of evidence will be classified into very low, low, moderate, or high judgment.

## 3 Discussion

According to a systematic review, KOA was ranked as the 11th highest contributor to global disability and 38th highest in disability-adjusted life years.^[17]^ Thus, effective intervention should be conducted in the management of KOA. In the consensus of 4-stepladder program of knee osteoarthritis (2018) in China, TCM was recommended as a complementary therapy for KOA for its function of relieving pain and enhancing mobility.^[18]^ Besides, several studies have reported that TCM can be utilized to treat KOA effectively.^[19,20]^ BSHX is a classical method of TCM in treating KOA. Currently, no systematic review has addressed the efficacy and safety of BSHX for the treatment of KOA. In this paper, we intend to conduct a systematic review and meta-analysis to evaluate the efficacy and safety of BSHX for KOA. We hope that this review will provide more reliable references to help clinicians make decisions when dealing with KOA.

## Author contributions

**Data curation:** Guocai Chen, Xiangling Ye.

**Formal analysis:** Guocai Chen, Xiangling Ye, Yingxin Guan.

**Investigation:** Guocai Chen, Xiangling Ye, Yingxin Guan.

**Methodology:** Guocai Chen, Xiangling Ye, Yingxin Guan.

**Project administration:** Wengang Liu, Jianping Du, Nan Yao, Xuemeng Xu.

**Review sponsor:** Xuemeng Xu.

**Software:** Guocai Chen, Xiangling Ye.

**Writing – original draft:** Guocai Chen, Xiangling Ye.

**Writing – review & editing:** Jianping Du, Wengang Liu.
